# Construction of Ag_3_PO_4_/g-C_3_N_4_ Z-Scheme Heterojunction Composites with Visible Light Response for Enhanced Photocatalytic Degradation

**DOI:** 10.3390/molecules29163774

**Published:** 2024-08-09

**Authors:** Xiangping Pan, Ying Meng, Qingwang Liu, Mai Xu

**Affiliations:** Anhui Engineering Research Center for Photoelectrocatalytic Electrode Materials, School of Chemistry and Material Engineering, Huainan Normal University, Huainan 232038, China; pxp_2005@126.com (X.P.); myhourse2001@126.com (Y.M.)

**Keywords:** Ag_3_PO_4_/g-C_3_N_4_, heterojunction, photocatalytic degradation, RhB

## Abstract

Ag_3_PO_4_/g-C_3_N_4_ photocatalytic composites were synthesized via calcination and hydrothermal synthesis for the degradation of rhodamine B (RhB) in wastewater, and characterized using X-ray diffraction (XRD), scanning electron microscopy (SEM), transmission electron microscopy (TEM), X-ray photoelectron spectroscopy (XPS), and diffuse reflectance spectroscopy (DRS). The degradation of RhB by Ag_3_PO_4_/g-C_3_N_4_ composites was investigated to evaluate their photocatalytic performance and cyclic degradation stability. The experimental results showed that the composites demonstrated notable photocatalytic activity and stability during degradation. Their high degradation efficiency is attributed to the Z-scheme transfer mechanism, in which the electrons in the Ag_3_PO_4_ conduction band and the holes in the g-C_3_N_4_ valence band are annihilated by heterojunction recombination, which greatly limits the recombination of photogenerated electrons and holes in the catalyst and enhances the activity of the composite photocatalyst. In addition, measurements of photocurrent (PC) and electrochemical impedance spectroscopy (EIS) confirmed that the efficient charge separation of photo-generated charges stemmed from strong interactions at the close contact interface. Finally, the mechanism for catalytic enhancement in the composite photocatalysts was proposed based on hole and radical trapping experiments, electron paramagnetic resonance (EPR) analysis, and work function evaluation.

## 1. Introduction

The utilization of nanomaterials in semiconductor photocatalytic degradation of organic pollutants offers high efficiency and low cost, making it a promising approach [1,2,3,4]. Recently, silver phosphate (Ag_3_PO_4_) has garnered significant attention for its outstanding photocatalytic performance in degrading organic pollutants under visible light irradiation [5]. Nevertheless, Ag_3_PO_4_ typically faces challenges such as high carrier complexity, severe photocorrosion, and a relatively large particle size, which notably restrict its photocatalytic performance [6,7,8]. In order to overcome these shortcomings and improve its photocatalytic efficiency and durability, the formation of a semiconductor heterojunction has been employed to enhance both the photocatalytic performance and stability of Ag_3_PO_4_.

It has been demonstrated that the heterojunction structure based on Ag_3_PO_4_ can effectively enhance the separation of photogenerated electron hole pairs, thereby promoting the generation of more reactive oxygen species [9,10]. This enhances the applicability of Ag_3_PO_4_ in solar photocatalysis. Therefore, constructing a Z-scheme is an effective method to enhance the photocatalytic activity of Ag_3_PO_4_ [11,12,13].

Graphitic carbon nitride (g-C_3_N_4_), a promising visible light-responsive photocatalyst, has been widely used in photoelectrocatalysis because of its stable chemical properties and remarkable responsiveness to visible light [14,15]. However, g-C_3_N_4_ is plagued by a wide band gap, high carrier complexity, and limited visible light response range [16,17]. The modification of g-C_3_N_4_-based photocatalysts can be investigated by expanding their light absorption range, which requires a narrower band gap. Furthermore, enhancing the acquisition or retention of electrons and holes with potent redox capability requires the presence of a more positive valence band and a more negative conduction band potential. Therefore, g-C_3_N_4_ forms heterojunctions with other semiconductors, but acquiring or retaining electrons and holes with potent redox capability demands a more positive valence band and a more negative conduction band potential. This not only broadens the light absorption range but also modulates the energy band structure to enhance the efficiency of charge-carrier separation. This results in a substantial reduction in the carrier complexation rate and an increase in the collection and utilization of visible light, significantly boosting the photocatalytic activity [18,19]. 

Constructing heterostructures for photocatalysts has emerged as a mainstream approach to enhance visible light utilization and expedite charge transfer and separation, thus overcoming the drawbacks of g-C_3_N_4_. Fewer factors are considered for constructing g-C_3_N_4_ heterojunctions [20] and the selected semiconductor generally only needs to have a suitably matched energy band structure. Based on the above analysis, the forbidden band widths of Ag_3_PO_4_ and g-C_3_N_4_ result in a staggered alignment, enabling the formation of an Ag_3_PO_4_/g-C_3_N_4_ heterojunction that demonstrates excellent photocatalytic activity under visible light irradiation [21,22,23].

Therefore, according to the previous research on Ag_3_PO_4_/g-C_3_N_4_ composites, the band structure of the semiconductor was determined by using the small angle valence band and tauc plots in combination with the Mott–Schottky curves. The direction of photogenerated carrier transfer was inferred from the work function, and further analysis focused on the catalytic degradation mechanism of the composites.

In this study, Ag_3_PO_4_/g-C_3_N_4_ heterojunction composites were prepared via simple calcination and hydrothermal methods. Simulated RhB solution was used to assess the degradation of organic pollutants and their role in RhB photodegradation. The experimental results confirmed that Ag_3_PO_4_/g-C_3_N_4_ composites exhibited high photocatalytic activity. Additionally, various advanced characterization techniques were used to characterize and analyze the Ag_3_PO_4_/g-C_3_N_4_ composites. Subsequently, a potential light-driven catalytic enhancement mechanism for Ag_3_PO_4_/g-C_3_N_4_ was proposed.

## 2. Results and Discussion

### 2.1. Structure and Microscopic Morphology of the Samples

**Figure 1:**
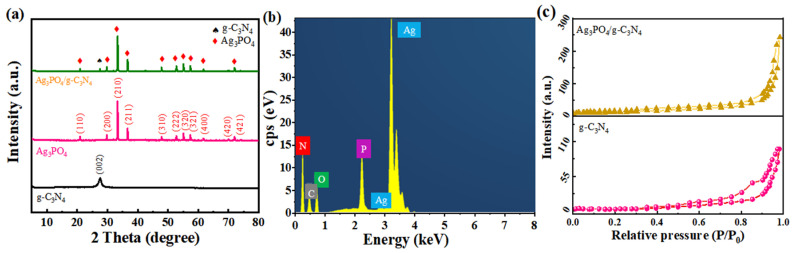
(**a**) XRD patterns of the different samples. (**b**) EDS pattern of the Ag_3_PO_4_/g-C_3_N_4_. (**c**) N_2_ adsorption and desorption isotherms of g-C_3_N_4_ and Ag_3_PO_4_/g-C_3_N_4_.

As can be seen from Figure 1a, two diffraction peaks of g-C_3_N_4_ appear at 2θ of 13.2° and 27.6°, corresponding to crystal planes (100) and (002), respectively [24,25]. The peak at 13.1° corresponds to the planar stacking structure of tri-s-triazine units within g-C_3_N_4_, while the diffraction peak at 27.6° is related to the interlayer stacking of graphite-like structures. These observations are consistent with previous research, confirming the successful preparation of graphitic phase carbon nitride. The 2θ of the diffraction peaks in the Ag_3_PO_4_ sample were 20.9°, 29.7°, 33.3°, 36.6°, 47.8°, 52.7°, 55.0°, 57.3°, 69.9°, and 71.9°, which corresponded to the diffraction peaks in the Ag_3_PO_4_ standardized atlas (PDF# 06-0505) of (110), (200), (210), (211), (310), (222), (320), (321), (400), (420), and (421) crystal planes. The absence of additional peaks confirms the high purity of the prepared Ag_3_PO_4_ [26,27]. The phase of Ag_3_PO_4_ remained unchanged in the composite catalyst Ag_3_PO_4_/gC_3_N_4_, whereas the (100) crystalline surface of g-C_3_N_4_ disappeared. This may be due to the low diffraction intensity of the (100) crystalline surface or the insufficient range of detection values [28]. It is noteworthy that the intensity of the Ag_3_PO_4_ diffraction peaks appeared reduced in Ag_3_PO_4_/g-C_3_N_4_, indicating that the introduction of g-C_3_N_4_ did not alter the material’s structure. The EDS energy spectrum in Figure 1b confirms the presence of Ag, P, C, N, and O elements in the sample.

In order to obtain the specific surface area of the prepared adsorbent materials, BET specific surface area tests were carried out on g-C_3_N_4_ and Ag_3_PO_4_/g-C_3_N_4_, and the results are shown in Figure 1c. Both g-C_3_N_4_ and Ag_3_PO_4_/g-C_3_N_4_ exhibited H_3_-type hysteresis loops in their N_2_ adsorption and desorption isotherms, characteristic of type IV isotherms. These observations were attributed to the stacking of Ag_3_PO_4_ particles and massive g-C_3_N_4_. This phenomenon can be attributed to the formation of slit holes in the Ag_3_PO_4_ particles and massive g-C_3_N_4_. The composite of g-C_3_N_4_ and Ag_3_PO_4_ exhibited a significantly increased specific surface area. An increased specific surface area enhances the availability of charge transfer channels and catalytic active sites.

**Figure 2:**
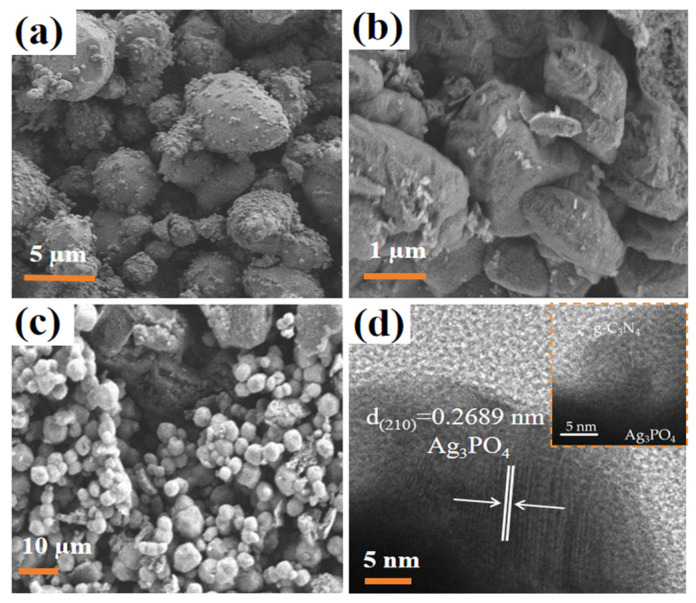
SEM images of (**a**) Ag_3_PO_4_, (**b**) g-C_3_N_4_, and (**c**) Ag_3_PO_4_/g-C_3_N_4_. (**d**) TEM images of Ag_3_PO_4_/g-C_3_N_4_.

The morphology of Ag_3_PO_4_ particles, illustrated in Figure 2a, appears irregularly spherical and agglomerated. As shown in Figure 2b, the pure g-C_3_N_4_ is an aggregated flocculent structure, which has the typical characteristics of g-C_3_N_4_ synthesized by thermal polymerization. When Ag_3_PO_4_ and g-C_3_N_4_ were combined (Figure 2c), their basic morphological features remain unchanged, resulting in a compact composite structure. In addition, Figure 2d reveals the close contact between Ag_3_PO_4_ and g-C_3_N_4_, forming a heterojunction with a crystal plane spacing of 0.2689 nm, which is consistent with the (210) crystal plane spacing of Ag_3_PO_4_. Notably, the high specific surface area of g-C_3_N_4_ facilitates enhanced adsorption of pollutant molecules during photocatalytic reactions. Simultaneously, the heterostructure formed between g-C_3_N_4_ and Ag_3_PO_4_ facilitates the separation of photogenerated electrons and holes, thereby enhancing the photocatalytic activity [29,30]. In summary, the successful construction of the photocatalytic composite Ag_3_PO_4_/g-C_3_N_4_ heterojunction is demonstrated.

### 2.2. XPS Analysis

**Figure 3:**
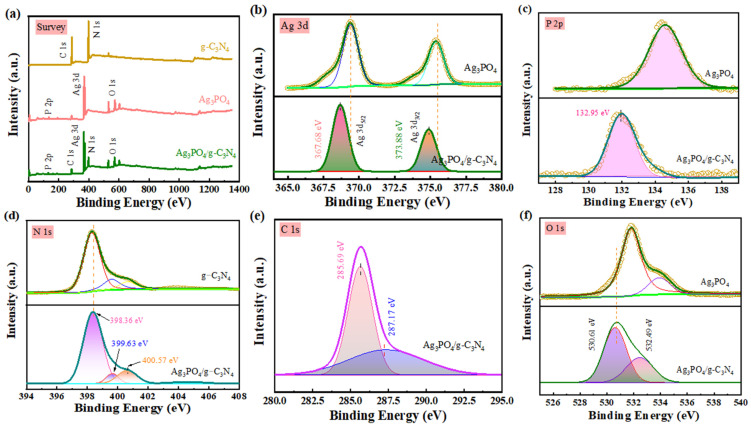
XPS spectra of the as-prepared samples. (**a**) The survey scan, (**b**) Ag 3d, (**c**) P 2p, (**d**) N 1s, (**e**) C 1s, and (**f**) O 1s.

The chemical composition and valence states of the various substances were determined using XPS measurements. Figure 3a presents the survey XPS spectrum of the Ag_3_PO_4_/g-C_3_N_4_ composite, revealing the presence of oxygen, silver, phosphorus, carbon and nitrogen. Figure 3b shows that the Ag 3d peaks with binding energies at 367.68 and 373.88 eV correspond to the Ag 3d_5/2_ and Ag 3d_3/2_ characteristic peaks, respectively, indicating the presence of silver in the composite photocatalysts [31,32]. As seen in Figure 3c, the peak of the P 2p spectrum at 132.95 eV can be attributed to the phosphorus in Ag_3_PO_4_. The N 1s XPS spectrum (Figure 3d) is decomposed into four peaks. The peak at 397.97 eV corresponds to the sp^2^ hybridized nitrogen (C-N=C) in the triazine ring due to the presence of different N-bonds in the repeating unit of the ring. The peaks at 398.85 and 400.57 eV are attributed to tertiary nitrogen N-(C)_3_ and terminal amino C-N-H, respectively [33,34]. The peak at the center of 404.62 eV is attributed to positive charge localization or charging effects in the cyano and heterocyclic rings or N-oxides. The C 1s spectra of Ag_3_PO_4_/g-C_3_N_4_ show two peaks (Figure 3e): 287.17 eV, corresponding to sp^2^ C atoms bonded to N (C-(N)_3_), and 285.69 eV, corresponding to amorphous carbon. Figure 3f displays the O 1s spectra. The peaks at 530.61 and 532.49 eV are attributed to the oxygen in the Ag_3_PO_4_ lattice and the surface oxygen species of the composites, respectively, including hydroxyl or carboxyl oxygen groups [35]. It is worth noting that after the combination of two single catalysts, the element binding energy of the composite material shifts compared to that of the pure catalyst, indicating a changed chemical environment and a strong interaction between Ag_3_PO_4_ and g-C_3_N_4_. Therefore, the composite material is not a simple physical mixture: Ag_3_PO_4_/g-C_3_N_4_ have achieved Z-scheme heterojunction.

### 2.3. Analysis of the Energy Band Structure

**Figure 4:**
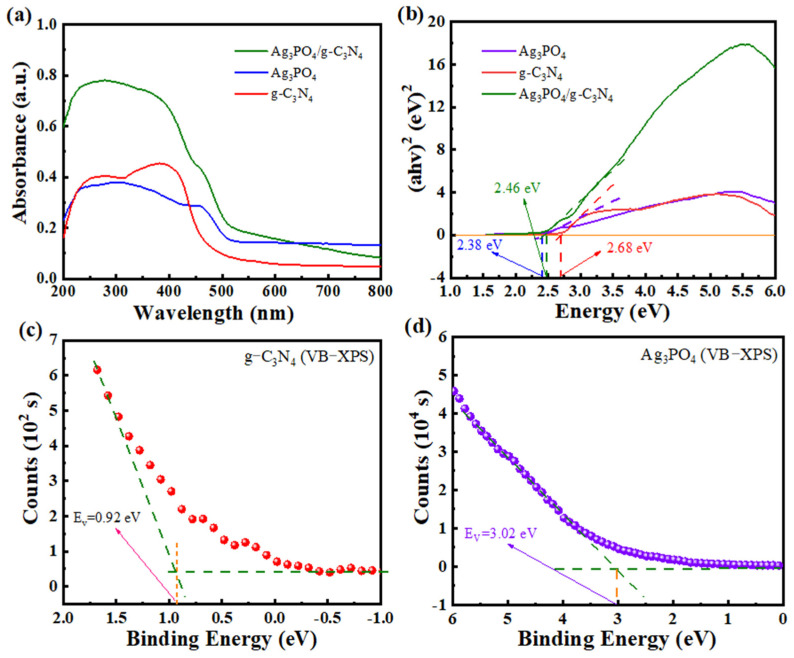
(**a**) UV–vis diffuse reflectance absorption spectra of samples. (**b**) Plots of (αhν)^2^ versus hν of samples. VB-XPS curves of (**c**) g-C_3_N_4_ and (**d**) Ag_3_PO_4_.

The light response characteristics of the samples were analyzed using UV–vis DRS. Figure 4a illustrates that the absorption intensity of the Ag_3_PO_4_/g-C_3_N_4_ composites changes in the visible range, exhibiting a broader range compared to pure g-C_3_N_4_. Furthermore, the addition of Ag_3_PO_4_ promoted the absorption of g-C_3_N_4_ in the visible range and improved its utilization, as evidenced by the red-shifted absorption bands observed in the composites [36,37]. Moreover, the forbidden band gaps of g-C_3_N_4_, Ag_3_PO_4_ and Ag_3_PO_4_/g-C_3_N_4_ are 2.68, 2.37 and 2.46 eV, respectively, as determined from the plot of (αhν)^2^ versus hν in Figure 4b. It is interesting to note that the forbidden bandwidth of the composites is narrower than that of g-C_3_N_4_, which suggests that the introduction of Ag_3_PO_4_ enhances the responsiveness of the photocatalysts to light and facilitates the absorption of lower energy photons.

Based on the VB-XPS plots of g-C_3_N_4_ and Ag_3_PO_4_ (Figure 4c,d), their VB-XPS potentials of g-C_3_N_4_ and Ag_3_PO_4_ are 0.92 and 3.02 eV, respectively. Therefore, using the formula:

$$E_{\mathrm{NHE}}=\varphi +E_{\mathrm{VB}-\mathrm{XPS}}-4.44$$

where E_NHE_ is the standard hydrogen electrode potential, φ is the electron work function of the XPS analyzer with a value of 4.55, and E_VB-XPS_ is the VB value tested by VB-XPS, the VB values of g-C_3_N_4_ and Ag_3_PO_4_ were calculated as 1.03 and 3.13 eV, respectively [38,39]. Subsequently, the conduction band (CB) values for g-C_3_N_4_ and Ag_3_PO_4_ were determined to be −1.65 and 0.75 eV, respectively, using the Nernst equation.

### 2.4. Optoelectronic Characteristics

**Figure 5:**
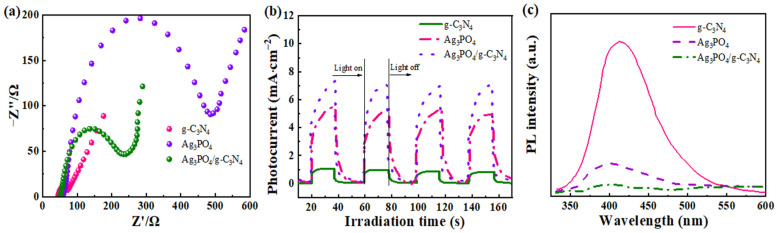
(**a**) EIS plots, (**b**) photocurrent density versus potential curves, and (**c**) PL spectra for g-C_3_N_4_, Ag_3_PO_4_, and Ag_3_PO_4_/g-C_3_N_4_.

Various techniques including electrochemical impedance spectroscopy, photocurrent testing, and photoluminescence are valuable for analyzing the efficiency of charge separation and transfer. Figure 5a illustrates that the charge transfer impedance of g-C_3_N_4_ is larger than that of Ag_3_PO_4_. This result is consistent with its weaker transient photocurrent results. Conversely, the impedance arc of the g-C_3_N_4_ composite with Ag_3_PO_4_ is significantly reduced, indicating lower charge transport resistance in the composite photocatalytic material. These findings suggest that the coupling of g-C_3_N_4_ with Ag_3_PO_4_ can effectively regulate the separation and migration efficiency of the carriers [40,41].

As depicted in Figure 5b, the individual photocurrent of g-C_3_N_4_ and Ag_3_PO_4_ is low under visible light irradiation conditions. However, when g-C_3_N_4_ and Ag_3_PO_4_ were composited, the composite material exhibited a significantly increased photocurrent density of 7.5 μA. This suggests that the successful construction of a heterojunction with g-C_3_N_4_ and Ag_3_PO_4_ effectively enhances the efficiency of photogenerated carrier migration [42,43]. This study investigated the separation efficiency of electron hole pairs in photocatalysts using the PL technique.

The PL spectra of g-C_3_N_4_, Ag_3_PO_4_, and Ag_3_PO_4_/g-C_3_N_4_ samples are presented in Figure 5c. Pure g-C_3_N_4_ exhibits a strong emission peak at approximately 425 nm, resulting from the complex reaction between electron and hole pairs. In contrast, the Ag_3_PO_4_/g-C_3_N_4_ sample exhibits the weakest PL signal, indicating significant suppression of the recombination of photoexcited carriers [44,45]. These findings suggest that the Ag_3_PO_4_/g-C_3_N_4_ composites are superior to g-C_3_N_4_ and Ag_3_PO_4_ samples in separating electron hole pairs, consequently enhancing the photocatalytic activity.

### 2.5. Evaluation of Photocatalytic Activity and Stability

**Figure 6:**
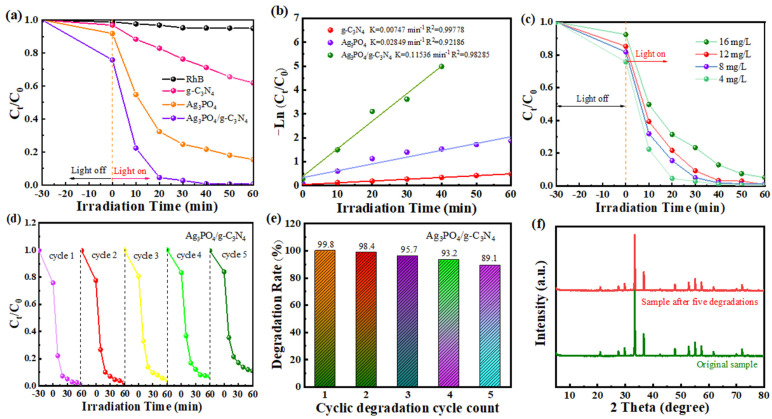
(**a**) Degradation curves of RhB by samples under visible light irradiation. (**b**) Corresponding first-order kinetics of samples. (**c**) The concentration effects of RhB on the photodegradation efficiency of the composite sample. (**d**,**e**) Five cycling runs of the composite sample for RhB degradation. (**f**) XRD patterns of Ag_3_PO_4_/g-C_3_N_4_ before and after the cyclic test.

The photocatalytic activity of the prepared samples was evaluated by degrading RhB solution under visible light irradiation. From Figure 6a, it can be seen that RhB self-degraded about 3% after 1 h of irradiation under visible light, indicating that the self-degradation of RhB is minimal. This indicates that RhB can be used as a target pollutant for evaluating photocatalytic degradation and catalytic activity. In the absence of light, all samples showed a certain degree of degradation, which might be caused by surface adsorption and photosensitization. While individual samples degraded a small amount of RhB solution, the Ag_3_PO_4_/g-C_3_N_4_ composite showed superior degradation performance. This enhancement could be attributed to the random stacking of nanosheets after composite formation, which increased the specific surface area.

It can also be seen from Figure 6a that the degradation rate of RhB by g-C_3_N_4_ changed minimally after 1 h of light exposure, achieving only 38%; in contrast, Ag_3_PO_4_ achieved 85%, and Ag_3_PO_4_/g-C_3_N_4_ notably reached 99.8%. This indicates that the visible light degradation efficiency of the composite photocatalytic material is significantly improved, which is mainly due to the narrower forbidden band width of the composite photocatalyst. It can better absorb and utilize the visible light, and its optical absorption characteristics are significantly enhanced. The heterojunction facilitates the separation and transmission of photogenerated carriers, enabling the composite material to effectively degrade organic pollutants. This leads to the highest photocatalytic degradation efficiency [46,47].

To further investigate the degradation performance of the composite photocatalysts, we investigated the kinetics of RhB photocatalytic degradation in the samples. The catalytic degradation process exhibited a one-stage reaction, as indicated by the −ln(C_t_/C_0_) versus time plot (Figure 6b). The photocatalytic apparent rate constants of the samples g-C_3_N_4_, Ag_3_PO_4_ and Ag_3_PO_4_/g-C_3_N_4_ were obtained as 0.00747, 0.02849 and 0.11536 min^−1^, respectively. Notably, the degradation rate constants of Ag_3_PO_4_/g-C_3_N_4_ were 15.48 and 4.06 times higher than those of g-C_3_N_4_ and Ag_3_PO_4_, respectively. 

The photocatalytic stability of the samples was evaluated by RhB photocatalytic degradation cycle experiments. Figure 6c displays the degradation efficiencies of Ag_3_PO_4_/g-C_3_N_4_ for various concentrations of RhB. The photocatalytic efficiency of the composites decreased with the increase in RhB concentration. In the RhB mass concentration of 16 mg·L^−1^ system, the degradation efficiency of RhB was maintained at about 95% within 60 min. In the RhB mass concentration of 12, 8 and 4 mg·L^−1^ system, an almost 100% degradation rate can be achieved within 60 min. Therefore, a high initial concentration of RhB is not conducive to the photocatalytic degradation process. This effect may stem from the high concentration of substrate in the reaction system, which diminishes light absorption and utilization by the material, thereby reducing the excitation energy of photogenerated electron hole pairs and hindering the photocatalytic performance of the system.

As shown in Figure 6d,e, the degradation rate of RhB by Ag_3_PO_4_/g-C_3_N_4_ remained above 89.1% after five cycles, demonstrating the high stability and reusability of the composite nanofiber material. This suggests its potential as a novel composite photocatalytic material for industrial wastewater purification. The XRD spectra of the composite photocatalysts before and after five degradation cycles are shown in Figure 6f. The intensity of the diffraction peaks of the samples did not decrease significantly, indicating that the crystal structure of the composite photocatalysts remained stable during the catalytic degradation process.

### 2.6. Photocatalytic Enhancement Mechanism

**Figure 7:**
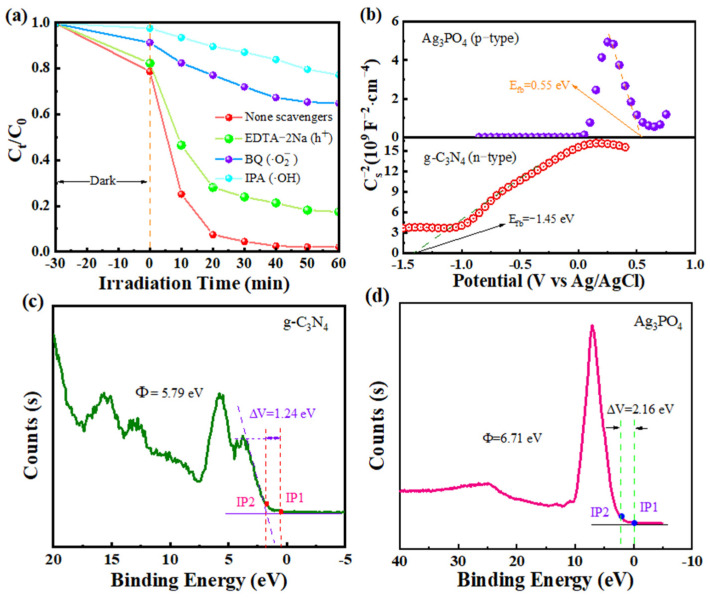
(**a**) Photocatalytic free radical capture degradation curves of Ag_3_PO_4_/g-C_3_N_4_. (**b**) Mott–Schottky curves of samples. VB-XPS curves of (**c**) g-C_3_N_4_ and (**d**) Ag_3_PO_4_.

The mechanisms of the primary active species involved in the photo-catalytic reaction of Ag_3_PO_4_/g-C_3_N_4_ were investigated through capture experiments. Figure 7a displays these results. The addition of BQ or IPA led to a decrease in the degradation rate of RhB from 99.8% to 35.1% and 23.9%, respectively. This indicates that ·OH and $\cdot O_{2}^{-}$ play crucial roles in the degradation of RhB. This result is plausible due to the E_CB_ value of −1.65 eV for g-C_3_N_4_, which facilitates the highly reductive nature of photogenerated electrons capable of reducing O_2_ to $\cdot O_{2}^{-}$ (O_2_/$\cdot O_{2}^{-}$(−0.33 eV vs. NHE)). Moreover, Ag_3_PO_4_ has an E_VB_ value of 3.13 eV (H_2_O/·OH (2.77 eV vs. NHE)) and can oxidize H_2_O to ∙OH. Adding EDTA-2Na did not reduce the breakdown of RhB much, indicating that h^+^ is not the main factor in this process.

In order to investigate the possible photocatalytic mechanism of the Ag_3_PO_4_/g-C_3_N_4_ heterojunction, the flat band potential of Ag_3_PO_4_/g-C_3_N_4_ was characterized. Mott–Schottky (M–S) curves of g-C_3_N_4_ and Ag_3_PO_4_ were examined at a frequency of 1000 Hz to determine their semiconductor properties. As shown in Figure 7b, the M–S curve of g-C_3_N_4_ exhibits a positive slope, indicating it is an n-type semiconductor. The M–S curve of Ag_3_PO_4_ showed a negative slope, confirming its p-type semiconductor nature. This suggests the formation of a p–n heterojunction between g-C_3_N_4_ and Ag_3_PO_4_. In addition, the flat-band potentials (E_fb,Ag/AgCl_) of g-C_3_N_4_ and Ag_3_PO_4_ measured at pH = 7, Ag/AgCl electrode conditions were −1.45 and 0.55 eV, respectively. These E_fb_ values were subsequently converted to the standard hydrogen electrode potential by the equation E_fb,NHE_ = E_fb,Ag/AgCl_ ± 0.197, from which the E_fb,NHE_ was calculated to be −1.65 and 0.75 eV for g-C_3_N_4_ and Ag_3_PO_4_, respectively [48,49]. Therefore, based on the above valence band values of g-C_3_N_4_-VB and Ag_3_PO_4_-VB (1.03 and 3.13 eV, respectively), the band gap of g-C_3_N_4_ and Ag_3_PO_4_ can be calculated by the formula: E_VB_ − E_CB_ = E_g_. The band gaps of g-C_3_N_4_ and Ag_3_PO_4_ are 2.68 and 2.38 eV, respectively, which is in agreement with Figure 4b.

Determining the work function difference between semiconductor materials is crucial for analyzing the direction of charge transfer at the material interface. Figure 7c,d depicts the work functions of g-C_3_N_4_ and Ag_3_PO_4_ obtained through small-angle valence band XPS spectroscopy (VB-XPS) measurements. When two semiconductor materials come into contact, electrons tend to migrate toward the material with the higher work function. Conversely, the material with the smaller work function tends to lose electrons until the Fermi energy levels of the two materials reach equilibrium. Consequently, the surface of the material with the lower work function becomes positively charged, while the surface of the material with the higher work function becomes negatively charged, creating a built-in electric field at the contact interface. According to the equation Φ = ΔV + φ (Φ is the work function of the sample, φ is 4.55 eV), ΔV is obtained from the distance between IP1 and IP2 (IP1 is the point of change of the binding energy with respect to the baseline and IP2 is the midpoint of the corresponding Fermi fringe curve) [50]. The distances between the two IP points of g-C_3_N_4_ and Ag_3_PO_4_ are calculated as 1.24 and 2.16 eV, respectively. Therefore, the work functions of g-C_3_N_4_ and Ag_3_PO_4_ are calculated as 5.79 and 6.71 eV, respectively.

**Figure 8:**
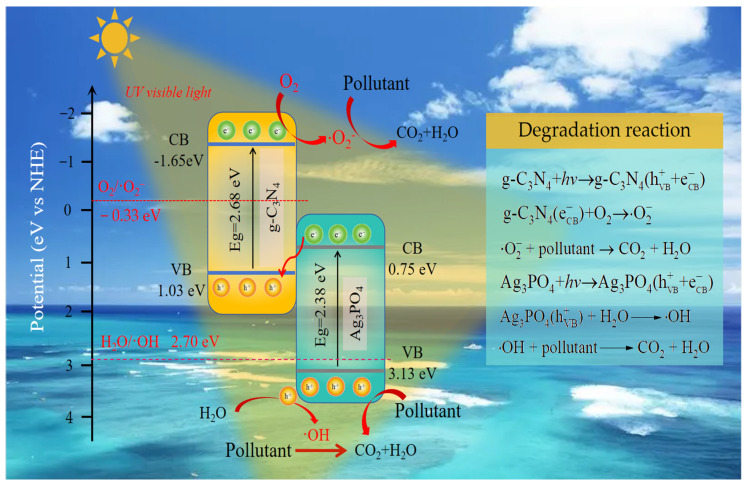
Schematic illustration of the degradation mechanism.

Theoretically, due to g-C_3_N_4_’s higher energy band edge and Fermi energy level compared to Ag_3_PO_4_, there is a high probability for the formation of Z-scheme complexes between these two materials. When the two semiconductors are in contact, the band edges in g-C_3_N_4_ continuously bend upward toward the interface, while the band edges in Ag_3_PO_4_ bend downward to the interface. As a result of the disruption of the charge transfer channel at the interface, photogenerated electrons from Ag_3_PO_4_ and holes from g-C_3_N_4_ may form complexes with each other instead of migrating into the energy band of the opposite semiconductor. Therefore, the electrons of g-C_3_N_4_ with high redox capacity and the holes of Ag_3_PO_4_ can be retained for the photocatalytic process, following the typical Z-scheme mechanism (Figure 8) [51,52,53].

## 3. Experimental Section

### 3.1. Reagents

Melamine (99% purity), silver nitrate (AgNO_3_), polyvinylpyrrolidone, disodium hydrogen phosphate dodecahydrate (Na_2_HPO_4_·12H_2_O), and rhodamine B (RhB) were purchased from Sinopharm Group Chemical Reagent Co. All chemicals were of analytical purity (AR) and were not further purified. Solutions were prepared using deionized water and used immediately.

### 3.2. Preparation of Samples

Typically, melamine (15.0462 g) was ground into a porcelain crucible, covered with a lid and heated to 550 °C for 4 h at a rate of 5 °C/min. After it was cooled to room temperature, the yellow-colored lumps of g-C_3_N_4_ were obtained, and the sample of g-C_3_N_4_ was obtained by grinding it into powder.

The g-C_3_N_4_ powder (0.1998 g) and AgNO_3_ (1.0010 g) were dissolved in 100 mL of deionized water. An amount of aqueous disodium hydrogen phosphate dodecahydrate (1.7899 g) and polyvinylpyrrolidone (1.2002 g) were added dropwise to the above solution under vigorous stirring. Stirring continued for 1 h, 150 mL of the solution was placed in a high-pressure reactor 200 °C constant temperature for 24 h. Then, the mixture was cooled to room temperature, filtered, and the resulting filtrate was washed three times with deionized water and then three times with ethanol. Finally, the filtrate was dried in a vacuum drying oven at 60 °C for 12 h. The sample was prepared as Ag_3_PO_4_/g-C_3_N_4_. The Ag_3_PO_4_ sample was synthesized in the same manner, except that g-C_3_N_4_ was not included.

### 3.3. Characterization of the Samples

The physical purity and crystal structure of the samples were examined using an X-ray diffractometer (XRD, Rigaku Ultima IV, Japan Co., Ltd., Tokyo, Japan). The morphology of the samples was investigated with a scanning electron microscope (FE-SEM, NANOSEM 450, FEI Co., Ltd., Hillsboro, OR, USA). Additionally, the samples were characterized by TEM using a high-resolution electron microscope (HRTEM, H-7000FA, Hitachi, Tokyo, Japan). A UV–visible spectrophotometer (UV–Vis DRS, UV-2550, Shimadzu, Japan, Kyoto) was used to characterize the energy band structure. Photoluminescence spectra were measured using a fluorescence spectrophotometer (PL, Jasco FP-6500, Tokyo, Japan). X-ray photoelectron spectroscopy (XPS, Axis Ultra) was used to collect data on the chemical composition and elemental valence of the sample surface. The samples’ photocurrent (PC) and electrochemical impedance (EIS) were measured using an electrochemical workstation (CHI660B). A photocatalytic device (ZQ-GHX-V, Zhenqiao, Shanghai, China) was used to test the photocatalytic performance.

### 3.4. Evaluation of Photocatalytic Performance and Stability

The photocatalytic activity of the samples was investigated through the application of a photocatalytic degradation process to rhodamine B. The samples (100 mg each) were dispersed in 50 mL of rhodamine B solution at a concentration of 4 mg·L^−1^. The adsorption equilibrium was reached after 30 min of dark adsorption. Photocatalytic degradation experiments were conducted at room temperature utilizing a 500 W xenon lamp with a 420 nm filter as the light source.

Samples were collected every 10 min. The supernatant was then centrifuged, filtered, and its concentration was determined using spectrophotometry. The stability of the photocatalysts’ degradation cycle was investigated through cyclic photocatalytic experiments. The photocatalytic activity was examined for five consecutive degradation experiments under the same conditions.

### 3.5. Cavity and Radical Trapping Experiments

Major active species during photocatalytic reactions were identified by hole and radical trapping experiments. Isopropanol (IPA) was used as the hydroxyl radical (·OH) trapping agent; p-benzoquinone (BQ) was used as the superoxide anion radical ($\cdot O_{2}^{-}$) trapping agent; and disodium ethylenediaminetetraacetate (EDTA-2Na) was used as the hole (h^+^) trapping agent. The relevant trapping agent (1 μmol·L^−1^) was added during the photocatalytic degradation reaction of RhB under the same conditions as those used for the evaluation of photocatalytic activity to obtain curves of RhB concentration versus time. The main active species of the photocatalytic reaction was determined by comparing the activity changes before and after the addition of the trapping agent.

## 4. Conclusions

In conclusion, an Ag_3_PO_4_/g-C_3_N_4_ heterojunction photocatalyst was prepared via a simple calcination and hydrothermal synthesis method to efficiently degrade RhB. The heterojunction between g-C_3_N_4_ and Ag_3_PO_4_ significantly enhances the separation efficiency and transport speed of the photogenerated carriers. The synthesized Ag_3_PO_4_/g-C_3_N_4_ composite photocatalysts exhibited significantly enhanced activity in RhB decomposition under visible light irradiation. Capture experiments indicated that the degradation process was primarily influenced by $\cdot O_{2}^{-}$ and ·OH, followed by H^+^. Finally, a possible Z-scheme degradation mechanism of organic pollutants by the Ag_3_PO_4_/gC_3_N_4_ composite photocatalyst is proposed based on the work function analysis and energy band theory. The composite photocatalyst exhibits high stability and reusability, making it a promising candidate for practical photocatalytic applications in industrial wastewater purification.

## Data Availability

Data are contained within the article.
